# A chronological model for the Late Paleolithic at Shuidonggou Locality 2, North China

**DOI:** 10.1371/journal.pone.0232682

**Published:** 2020-05-27

**Authors:** Fei Peng, Sam C. Lin, Ilaria Patania, Vladimir Levchenko, Jialong Guo, Huimin Wang, Xing Gao

**Affiliations:** 1 Department of Archaeology and Museology, School of Ethnology and Sociology, Minzu University of China, Beijing, China; 2 Key Laboratory of Vertebrate Evolution and Human Origins of Chinese Academy of Sciences, Institute of Vertebrate Paleontology and Paleoanthropology, Chinese Academy of Sciences, Beijing, China; 3 Centre for Archaeological Science, School of Earth, Atmospheric and Life Sciences, University of Wollongong, Wollongong, New South Wales, Australia; 4 Australian Research Council Centre of Excellence for Australian Biodiversity and Heritage, University of Wollongong, Wollongong, New South Wales, Australia; 5 Zinman Institute of Archaeology, Haifa University, Haifa, Israel; 6 Centre for Accelerator Science, Australian Nuclear Science and Technology Organization, Lucas Heights, New South Wales, Australia; 7 Institute of Culture Relics and Archaeology of Ningxia Hui Autonomous Region, Yinchuan, China; 8 Chinese Academy of Sciences Center for Excellence in Life and Paleoenvironment, Beijing, China; 9 University of Chinese Academy of Sciences, Beijing, China; Universita degli Studi di Milano, ITALY

## Abstract

The site of Shuidonggou Locality 2 offers important evidence for the Late Paleolithic sequence of north China. The site not only contains one of the earliest instances of ornamental freshwater shell and ostrich eggshell beads in the region, but also stone artifacts with features arguably resembling the Initial Upper Paleolithic (IUP) blade technology found farther north. The appearance of these innovative archaeological forms have been attributed to the arrival of hominin populations, possibly modern humans, into the region during Marine Isotope Stage 3. Yet, the chronology of the site remains debated due to ambiguities in the existing dates. In this study, we conduct a systematical radiocarbon analysis of charcoal and ostrich eggshell samples obtained throughout the site sequence. Both acid-base-acid and the more stringent acid-base-oxidation pretreatment methods were applied to the charcoal samples. The resulting ages follow an age-depth relationship that is consistent with the stratigraphic profile. In line with previous stratigraphic assessments, Bayesian age modeling suggests that site formation history can be split into two phases: an early phase 43–35 cal kBP associated with a lacustrine depositional environment, and a later phase 35–28 cal kBP associated with rapid terrestrial silt accumulation. The chronology of the archaeological layers containing IUP-like artifacts are placed at 43–39 cal kBP and 35–34 cal kBP respectively. This finding supports the interpretation that an IUP-like blade technology appeared in the SDG region by at least ~41 ka.

## Introduction

The general impression of the Chinese Paleolithic is one of long-term technological simplicity and stasis, marked by the persistence of core-and-flake and cobble-tool industries in North and South China respectively over much of the Pleistocene [1,2; but see 3]. By ~40–30 thousand years (ka) ago, however, the Paleolithic record of North China witnessed abrupt changes with the appearance of material evidence indicative of innovative technology (e.g., blade and microblade production) and symbolism (e.g., ornaments)—traits commonly associated with modern human behavior. This shift, which has been referred to as the onset of the Late Paleolithic in Chinese Paleolithic studies [1,4], has been viewed to reflect the spread of humans, potentially *Homo sapiens*, into this part of the world during Marine Isotope Stage (MIS) 3 (60 to 27 ka), and their possible interaction with existing human groups in the region [5–10].

**Fig 1:**
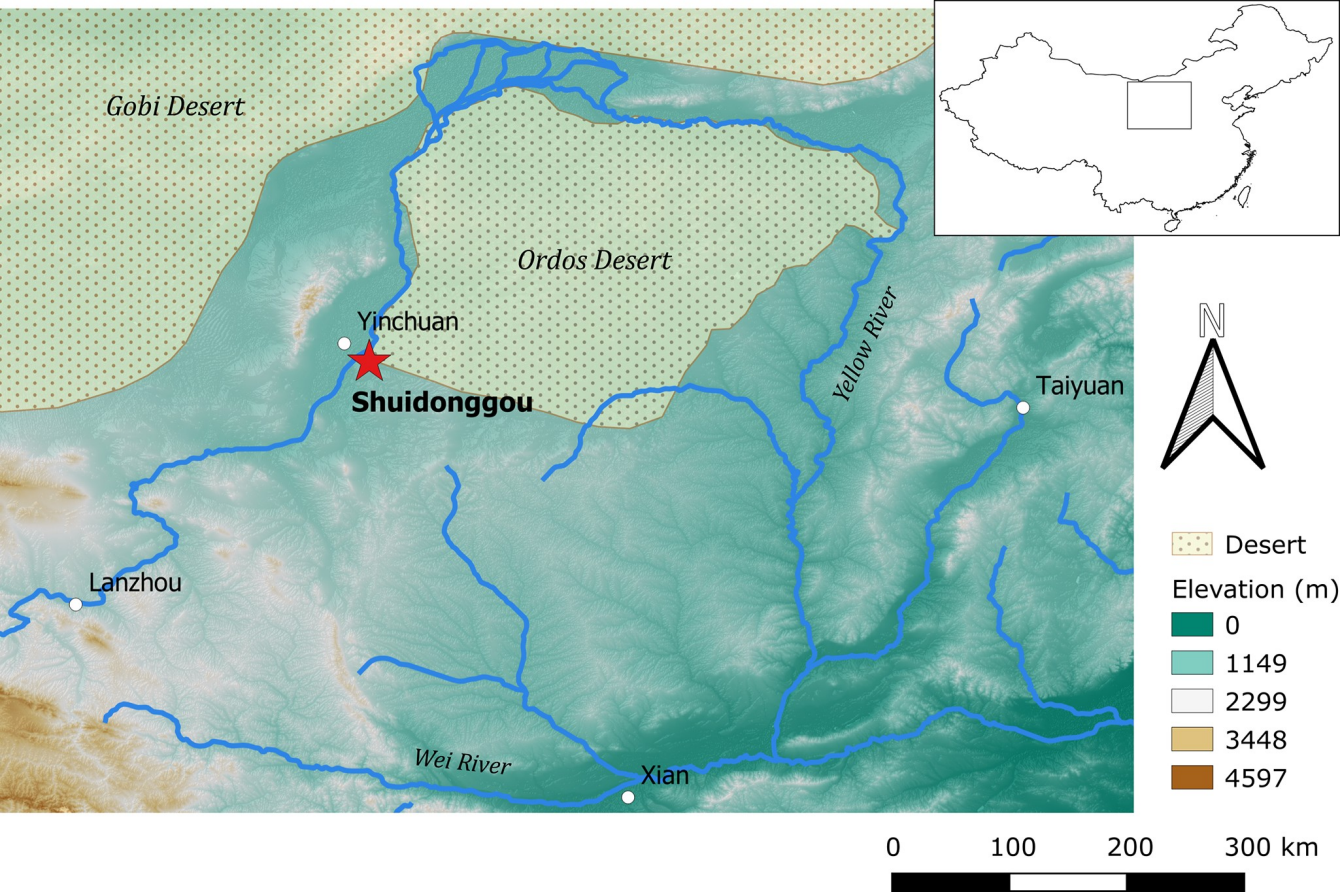
Location of Shuidonggou. Red star indicates the location of the Shuidonggou site complex (38°17´55”N, 106°30´6”E). Produced with data in the public domain from Natural Earth (https://www.naturalearthdata.com/) and the U.S. Geological Survey Earth Resources Observation and Science (EROS) Center (https://www.usgs.gov/centers/eros). The elevation model used is the USGS EROS Shuttle Radar Topography Mission (SRTM) Void Filled Digital Elevation [17].

A primary example of the Late Paleolithic in North China is Shuidonggou (SDG). Located at the edge of the Ordos Desert in the Ningxia Hui Autonomous Region (Fig 1), SDG is a cluster of 12 open-air localities with concentrations of archaeological materials preserved within loess-like sediment deposits. Since its initial discovery in 1923, SDG has received much research attention due to the unique Pleistocene lithic assemblage recovered from Locality 1 (SDG1) that contains a mixture of Levallois and blade technologies [11,12]. Today, researchers generally agree that the SDG1 lithic assemblage is comprised of a large blade technology associated with the so-called Initial Upper Paleolithic (IUP) found farther north in Mongolia and Siberia 47–30 ka [13,14]. Defined by its lithic characteristic that involves a mixture of Middle Paleolithic Levallois reduction and Upper Paleolithic volumetric core exploitation, the IUP occurred from 50 ka to 35 ka across a large geographic area, ranging from the Levant to Eastern Europe, Siberia, Mongolia and China [15,16]. At these places, the IUP represents the earliest form of the Upper Paleolithic [15], marking a clear archaeological change in lithic technology and other material culture, including the appearance of bone tools and symbolic materials such as beads, engraved objects and perforated pendants at some sites.

The specificity of the IUP lithic technology has been argued to support the notion that the IUP represents a coherent techno-complex, produced possibly by a common culture that dispersed across Eurasia towards the end of the Pleistocene [15,16]. Yet, it remains open to question which hominin group(s) was/were responsible for the IUP as very few hominin fossils have been found in clear association with IUP assemblages to date [15]. Given recent findings about the diversity of late hominin populations in central/east Eurasia and their capacity for symbolic expression and innovative technology [18–20], both Neantherthals and the Denisovans represent plausible candidates for the IUP in addition to *H*. *sapiens* and other possible archaic hominins [15].

However, in Northeast Asia, researchers have generally attributed the IUP in the region to the spread of modern humans to east Asia [21–23]. Indeed, the early appearance of the IUP ~45 ka at the site of Tolbor 16 in Mongolia coincides with the earliest evidence of modern humans in the Eurasian Steppe [21]. This time period also coincides with the first occurrences of symbolic ornaments and bone tools in the region’s archaeological record [16,24]. Based on available dating results of IUP sites in Northeast Asia, researchers have postulated that the IUP in this region originated in the Altai ~47 ka [16,19] and spread eastward to the Baikal region, northern Mongolia, southern Mongolia and North China around 45 ka [21]. Others have suggested an alternative route across the Altai and Tian Shan Mountains, through to the Gobi Desert and North China [25]. Differentiating between these two IUP dispersal paths will help clarify the nature and evolution of the IUP, and potentially shed important light on the movement and dispersal of modern humans into East Asia. Yet, establishing the timing and pathways of the IUP spread is currently difficult due to ambiguities in age estimates, particularly at sites in China that contain materials with IUP features [15].

### Dating the IUP at Shuidonggou

Being the southernmost example of the IUP phenomenon in North Asia, the chronology of SDG is critical for clarifying the order and direction of the IUP spread [8]. Yet, despite numerous dating efforts, the chronology for the IUP at SDG remains debated [26,27]. For SDG1, existing understandings of the site chronology is plagued by inconsistent results and equivocal contextual association. For example, a number of the dates commonly cited for the Pleistocene deposit of SDG1 were published more than three decades ago [28–30] and their quality are difficult to assess today. More recently, Liu et al. [31] dated the site using Optically Stimulated Luminescence (OSL) analysis. Out of the seven sediment samples analyzed from the Pleistocene layer, five produced a consistent age-depth sequence in the 29–36 ka range, but other two samples intermitted in this sequence yielded significantly younger results (16 ka and 18 ka).

In 2014, a radiocarbon date of 36,200±140 ^14^C BP derived from a charcoal sample collected from the Pleistocene profile of SDG1 was published by Peng et al. [32]. This age was calibrated at 2σ to 41,009–41,728 cal BP by Morgan et al. [8] and 39,323–39,227 cal BP by Li et al. [33] using different software and calibration curves. Yet, because the charcoal sample was not obtained during excavation but instead from an exposed and highly eroded profile, the association between this age estimate and the IUP context at SDG1 remains uncertain [15]. The most recent systematic dating study of SDG1 was by Nian et al. [34] using single aliquot OSL dating. They dated samples from the Pleistocene context at two separate areas of the currently exposed stratigraphic profile: the north section and the southwest section. The resulting ages place the three dated stratigraphic unit at 46–43 ka, 39–33 ka, and 22 ka respectively. While the authors noted that the chronology of these units are in stratigraphic order, the samples dated to 39–33 ka (which were collected from the southwest section) are actually lower in elevation than the samples dated to 46–43 ka (which were obtained from the north section). The reason for this elevation difference in the stratigraphic sequence between the two sections remains unclear.

Alternatively, other researchers have examined the timing of the IUP in North China by dating IUP-like materials from other sites in the region. For instance, at the South Temple Canyon site, 80 km northwest of SDG, Madsen et al. [7] identified a limited number of stone artefacts that contain technological resemblance to the SDG large blade technology. By dating the carbonate crust on a stone flake associated with these blade artefacts, they reported a radiocarbon age of 41,070±890 ^14^C BP, which calibrates to ~46–43 ka cal BP at 2σ in OxCal 4.3 [35] using the IntCal 13 calibration curve [36]. This age estimate suggests an early spread of the IUP to north China from the Altai through western and central Mongolia. However, while this age estimate may be sound, these stone artefacts were derived from a non-excavation setting, and the stone flake that was analyzed does not itself possess any blade technological features. As such, the contextual association between the dated stone flake and the IUP phenomenon is open to question.

Additional IUP-like lithic materials have been identified at SDG in an excavation context at Locality 2, 7 and 9 (SDG2, 7 & 9) [33,37,38]. Levallois-like cores were recovered at SDG7, which is located ~300 m southeast of SDG1, from a layer dated to 30 ± 3 ka–23 ± 2 ka by OSL [38]. At SDG9, ~7 km southeast of SDG1, excavation recovered IUP-like lithic materials including a bidirectional Levallois blade core, a narrow-faced volumetric blade core, Levallois flakes with faceted platforms, and a rejuvenation core tablet [37]. A single OSL age places the layer from which the lithics came from to ~36–27 ka [5,37], but the excavators cautioned against the use of this age estimate at face value because the archaeological deposit sat relatively close from the modern-day surface (~80 cm in depth). Unfortunately, SDG9 has since been destroyed by construction and thus it is impossible to verify this chronology today.

#### Shuidonggou Locality 2: Previous research

SDG2 is ~150 m southeast of SDG1; the two sites are separated by a small tributary of the Yellow River. Prior to the excavation of SDG2, Madsen et al. [23,39] described a Levallois core with bidirectional convergent removals and a faceted platform collected from one of several naturally exposed hearth features at the site. Radiocarbon analysis on charcoal and eggshell samples derived from the hearth features returned eight dates between 29–24 ka ^14^C BP, which were calibrated to ~34–28 ka cal BP by Li et al. [33] using OxCal 4.1 [35] and the IntCal 09 calibration curve [40]. In 2003–2007, the Institute of Vertebrate Paleontology and Paleoanthropology and the Institute of Culture Relics and Archaeology of Ningxia Hui Autonomous Region carried out extensive excavation at the site [41–43]. The excavation took place at two adjoining areas, T1 and T2, and exposed a stratigraphic sequence that is over 12 m thick [see 23,34 for detail]. Eighteen geological layers were identified on the basis of sedimentary characteristics and the presence of archaeological remains [31]. The lower layers are comprised of green silty clay and dark, organic rich sediment with clear lamination and bedding, indicating a lake or marsh environment during the early phase of the site formation history [31]. The upper layers are composed almost entirely of massive yellow silt deposits with some lamination and aragonite ooids bands. The change in the sediment between the upper and lower layers indicates a shrinkage of the previously occurring waterbody at the site. The lake-related sedimentary history highlighted above is supported by the pollen record, which indicates a semi-arid climate with the presence of wetland bodies on the landscape [31].

Archaeological remains including stone tools, animal bones and ostrich egg shell fragments occur throughout much of these geological layers [43,44]. Based on the concentration of artifacts and their correlation with the geological layers, the excavators classified these artifacts into seven cultural layers (CL1–7) [31,33]. The upper five cultural layers (CL1–5) are comprised of concentrations of artifacts organized in horizontal bands separated by sediment containing sparse to no archaeological remains. These five cultural layers occur in the top ~6 m of the stratigraphic sequence and are associated exclusively with the loess-like yellow silt deposits. The lower cultural layers (CL6–7), on the other hand, are made up by artifacts loosely distributed in lower quantities among the lower-most part of the yellow silt deposit and the green/black silty clay sediment body below.

The hearth features from which IUP-like materials were identified previously by Madsen et al. [23] at the site were attributed to CL2 [45]. Yet, curiously, no lithic materials with IUP features were found in CL2 during excavation. In fact, the lithic materials recovered from the site are characterized by informal core-and-flake materials [46], except for two cores from the lower CL5 and CL7 that have been identified that contain features indicative of a IUP-like large blade technology [33].

The IUP-like cores at SDG2 provide a tentative indication for the occurrence of a large blade technology at the site akin to that observed at SDG1 and SDG9. The deep stratified sequence of SDG2 also provides an important source of information for clarifying the chronology of the nearby SDG1 and the general timing of the IUP in north China.

**Fig 2:**
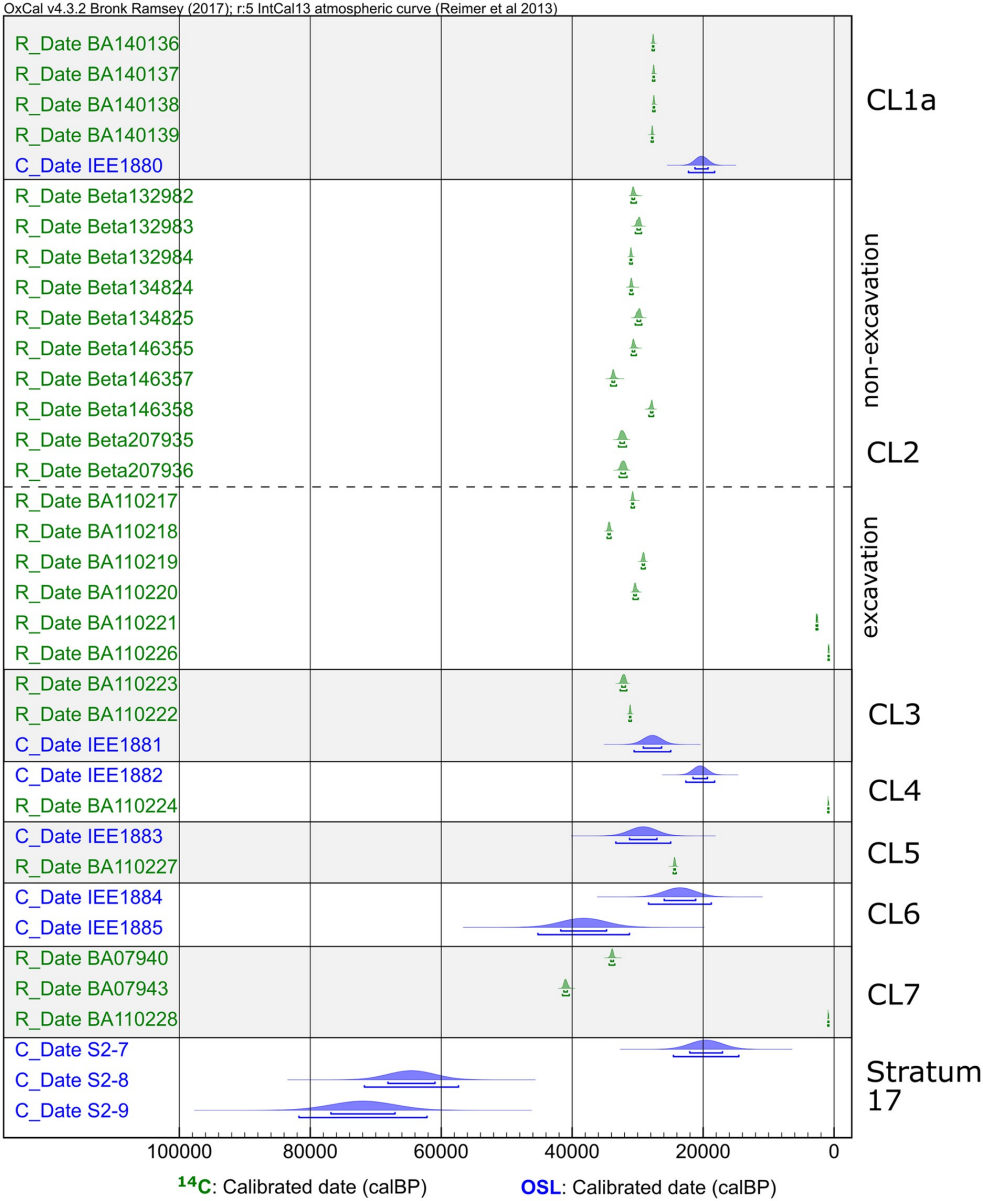
Published dates from SDG2. Dates are plotted by their associated Cultural Layer or stratigraphic layer. Within each layer unit, however, the ordering of the samples follows their laboratory ID sequence and does not reflect relative depth. This is because the depths of these samples were not recorded during sample collection.

Several studies have dated the site since its excavation [31,33,45,47], and a total of 33 age estimates (^14^C and OSL) have been produced from excavated samples (including those reported by Madsen et al. [23]) (see S1 Table). These published ages are summarized in Fig 2 using OxCal 4.3 [35]. The ^14^C dates are calibrated against IntCal13 [36] with the *R_Date* command; the OSL ages are corrected for their year of publication and then plotted using the *C_Date* command, which specifies a normally distributed likelihood (see S1 Appendix for OxCal code). These ages generally place the SDG2 archaeological deposit between 41 and 27 k calendar years BP, and the timing for the IUP-like cores identified by Li et al. [33] was placed between ~41 and 34 k calendar years BP. However, these chronologies are contentious due to notable discrepancies among the dates. Aside from the presence of extremely young dates (<3 ka) that are likely outliers due to contamination, the most notable issue is a lack of a clear age-depth correlation [26]. For instance, the ^14^C dates from excavated samples in CL2–3 range in 35–28 ka, but the underlying CL4–5 were dated to 29–21 ka by OSL and 24 ka cal BP by ^14^C. There are also inconsistencies among dates derived from the same stratigraphic unit. The two OSL ages from CL6 yielded markedly different ages of 38 ka and 24 ka, while the lowermost CL7 produced ^14^C ages of 41–40 ka and 34–33 ka cal. BP; the sterile unit under CL7 was dated by OSL to 20 ka, 65 ka and 72ka. Recently, Li et al. [47] conducted a ‘chronological hygiene’ assessment of the existing SDG2 ages and rejected 16 of the published dates that either are clear outliers with extremely young dates or do not fit with the stratigraphic sequence.

Although the reason for the incongruities among the dates is unclear, the issue may be due to the fact that these ages were produced on different kinds of samples by different techniques and laboratories. For instance, the ^14^C ages for CL2 were measured by Beta Analytics and the Institute of Heavy Ion Physics at Peking University, while the remaining radiocarbon dates from CL3–5 and CL7 were produced solely by the Institute of Heavy Ion Physics of Peking University. There is also limited information about the analytical procedures underlying these age estimates, such as the pretreatment process and quality control measures [e.g., δ (_13_C), % of modern carbon] for the ^14^C samples. Moreover, the provenience of the dated samples was limited to the excavation spit and associated CLs; their actual spatial position and depth were not recorded. It is therefore difficult to evaluate whether issues in the site chronology are related to stratigraphic association of dating samples. Considering these factors, the chronology of SDG2 remains inconclusive and needs to be re-evaluated.

To help resolve this issue, here we report a systematic dating study of SDG2 conducted as part of a renewed field research project of the site. Using a combination of conventional and more stringent pretreatment methods, our results show smaller errors in comparison to the previous dates and a good age-depth relationship along the stratigraphic sequence. Bayesian modeling suggests the site was part of a lake system 43–35 ka cal BP before transitioning to a terrestrial, possibly lake shore environment with periodic human occupation ~35–28 ka cal BP. Our chronology places the timing of first appearance of the large blade cores at SDG2 around 43–34 ka cal BP, supporting the general age estimate proposed by Li et al. [33] and Morgan et al. [8] for the probable occurrence of the IUP at SDG as early as ~41 ka. This chronology puts the IUP in north China slightly younger than those in Altai, Mongolia and the Baikal region farther north and west, and suggests a rapid eastward and southward expansion of the IUP phenomenon from the Altai ~47–41 ka.

## Material and methods

**Fig 3:**
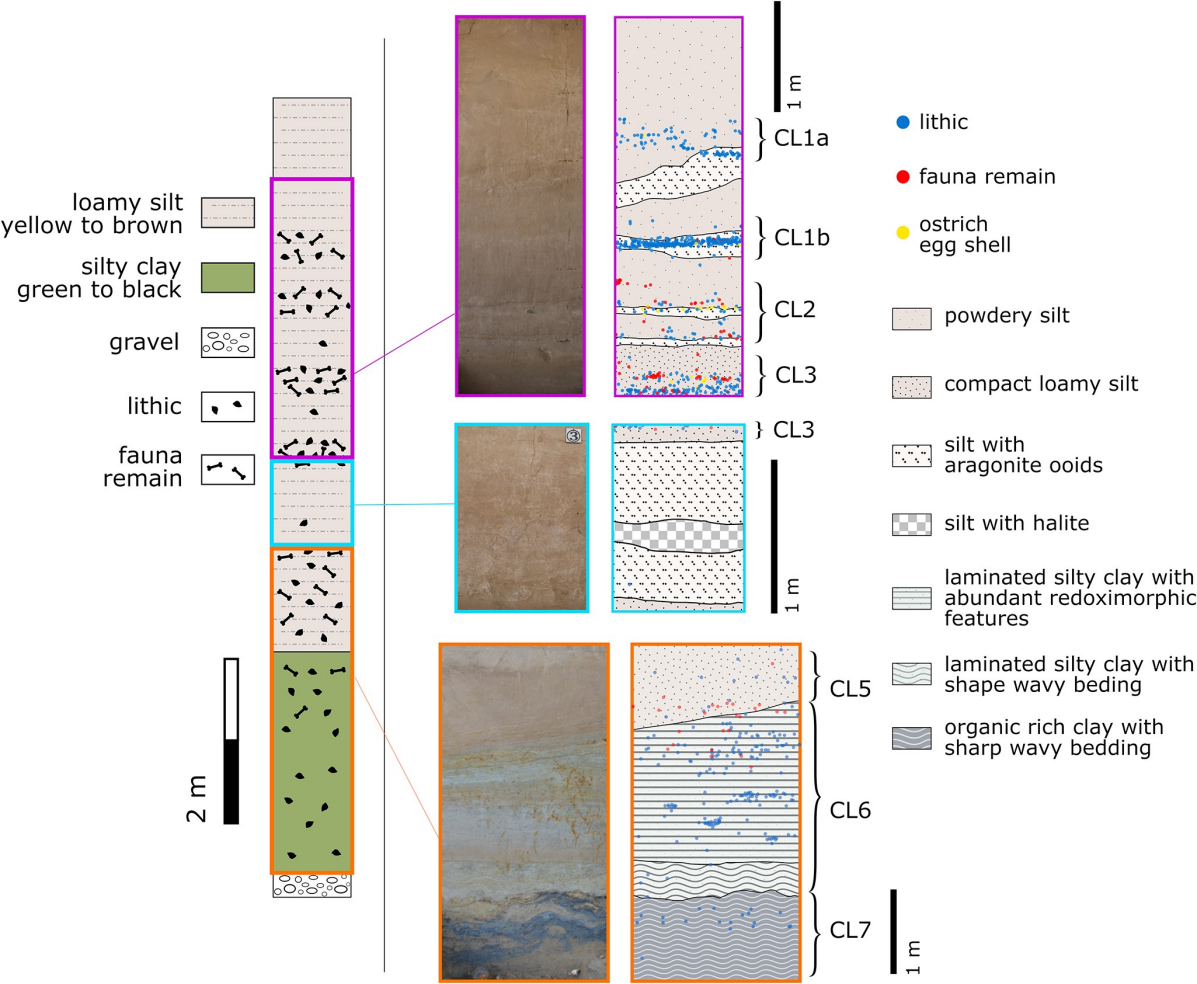
SDG2 T3 stratigraphy. On the left, the schematic column summarizes the general sediment characteristic and archaeological remain distribution of the site stratigraphic sequence. On the right, the photographs and schematic drawings show a close-up view of different parts of the stratigraphic sequence. The distribution of the three main types of archaeological remains recovered (lithics, fauna remains and ostrich egg shell fragments) corresponding to the photographed sections are overlain on the schematic drawings. The partitioning of the seven cultural layers (CL1a–7) are denoted beside the schematic drawings.

In 2014–2016, a new excavation at SDG2 was carried out with a permit provided by the National Cultural Heritage Administration of China (permit number 2014–359, 2015–210, 2016–230) to X Gao who is the leader of this excavation. All necessary permits were obtained for the described study, which complied with all relevant regulations. The excavated area (T3) is adjacent to the part of the site exposed by the previous excavation (T1 & T2) [48]. The excavation exposed a 10 m stratigraphic sequence akin to the one reported previously at T1 and T2. The upper ~7 m of the sequence is composed of micro-laminated loess-like yellow silt with localized high halite content and horizontal bands of aragonitic ooids (Fig 3). These deposits were likely formed under a low-energy water environment, such as in a fluctuating lakeshore setting. The lower part of the sequence is related to a lake or marsh environment and made up of laminated green silty clay lake deposits on top of dark, organic rich clay with sharp wavy bedding. The laminated green silty clay layer, which contains abundant redoximorphic features, is separated from the yellow loamy silt layer above by a diffused bedding boundary of laminated compact loamy silt to clay silt.

**Fig 4:**
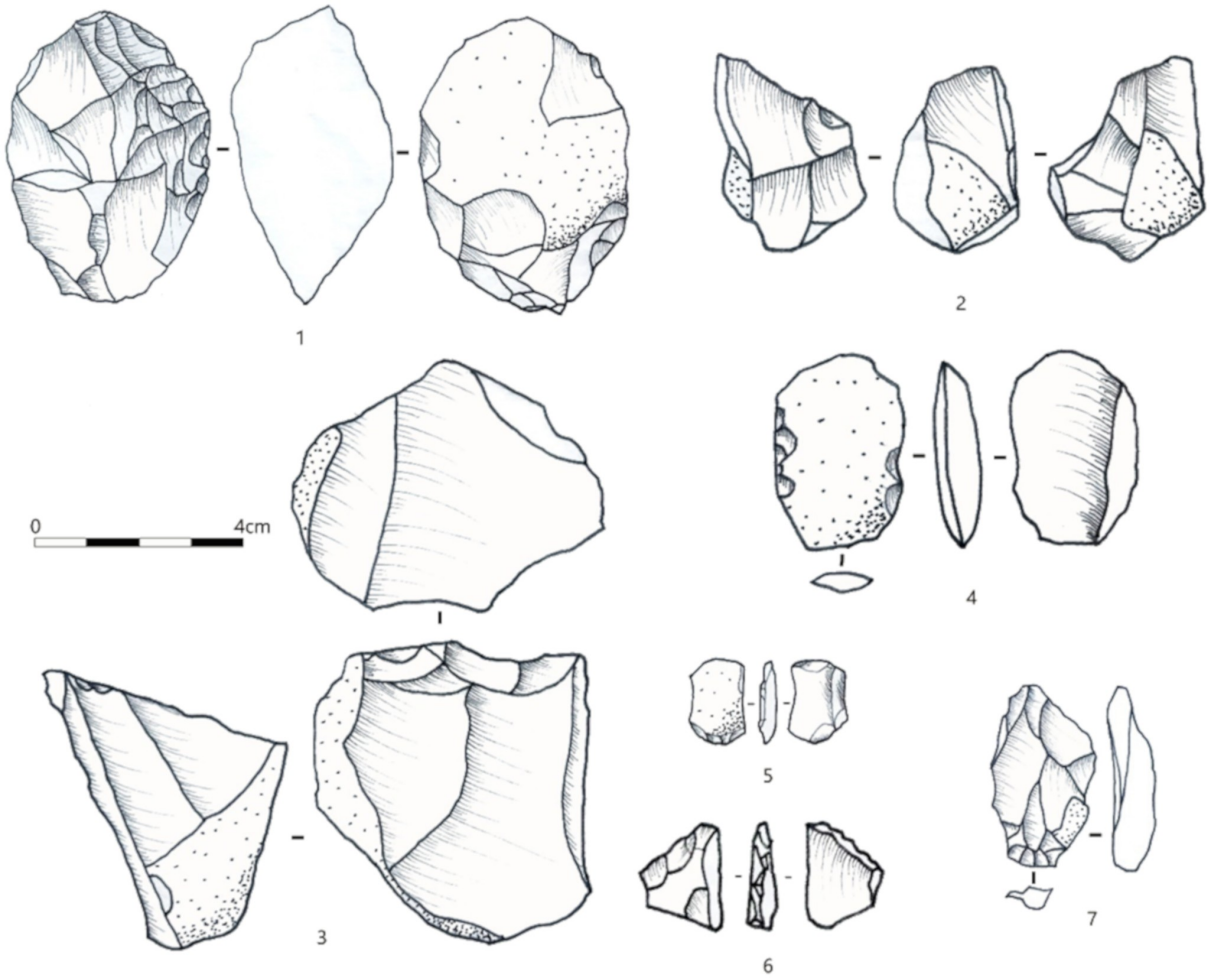
Examples of stone artifacts from T3, SDG2. 1–3 cores, 4–6 retouched pieces, 7 flake.

Except for CL4, all the cultural layers identified in the previous excavation were recognized at T3 with similar characteristics (Fig 3). Namely, the upper CL1–5 (with CL1 subdivided in CL1a and CL1b) are comprised of concentrations of artifacts organized in horizontal bands that occur on the top half of the stratigraphic sequence. The lower CL6–7, defined on the bases of stratigraphic facies, contain lower quantities of artifacts distributed loosely among the sediment body. More than 5000 stone artifacts with maximum dimensions >20 mm were excavated from T3. Similar to the previous excavation, these lithic materials are composed mainly of informal artifacts made on locally abundant stone types (e.g., siliceous limestone, quartzite, chert) with no clear technological patterning of standardized production [49–51] (Fig 4).

In terms of taphonomy, there are no clear signs of post-depositional processes that may have influenced the distribution of the archaeological finds. Orientation of elongated lithics and bones show a randomly distributed bearing alignment among the cultural layers (Li et al. in prep), suggesting the assemblages were unlikely to have been transported by taphonomic factors such as water flow. While there are some signs of bioturbation indicating grass root activities and small insect burrowing, the extent is minimal and localized. The overall preservation of organics at the site is poor. Most of the recovered bones are in small fragile fragments that would disintegrate when washed by water. When observed in situ, bones show a gray (possibly calcitic) halo impregnating the sediments immediately around it, suggesting a chemical diagenesis rather than mechanical.

Samples for radiocarbon dating analysis were systematically collected throughout the stratigraphic sequence during the excavation. Due to a lack of clear stratigraphic boundaries throughout the sequence, dating samples were obtained in association with various cultural layers. Specifically, for the upper four cultural layers (CL1a, 1b, 2, 3), samples were selected from materials that occur in clear association with the densely concentrated horizontal bands of artifacts. For the lower three cultural layers (CL 5, 6, 7) that are defined more on the bases of sediment characteristics, dating samples were collected from the geological layers associated with the cultural layers. Charcoal occur throughout the sequence but mostly as isolated, floating specks (millimeter sized) within the sediment deposit. In this context, our sampling strategy prioritized charcoals from unequivocal combustion features where possible, as well as larger isolated charcoal and ostrich eggshell fragments. Samples were collected away from the edge of the excavation area that may have been exposed in the past or affected by bioturbation. The genus and species of the charcoal samples were not identified due to their small sizes. However, unpublished preliminary archaeobotanic work suggests that the charcoals represented at T3 are primarily derived from short-lived plant groups, including subfamily *Chenopodiaceae*, genus *Salix*, *Elaeagnus angustifolia*, genus *Tamarix*, and genus *Populus*.

A total of 32 samples were selected to evenly represent the CLs along the stratigraphic sequence. The lower units, particularly the geological layers associated with CL6–7 and the basal gravel layer have few archaeological remains in general, thus the number of samples from these contexts are limited. Because the contact boundary between the stratigraphic layers associated with CL5 and CL6 is slopped (as shown in Fig 3), additional samples were chosen for CL5 to clarify the stratigraphic boundary with the underlying CL6. These samples were submitted to the Australian Nuclear Science and Technology Organisation (ANSTO) for radiocarbon analysis, with an import permit provided by the Department of Agriculture and Water Resources of the Australian Government (permit no. 0000664336). We employ the more stringent Acid-Base-Oxidation, or ABOX [52,53] pretreatment, on larger charcoal samples; whereas the conventional Acid-Base-Acid (ABA) pretreatment is applied to smaller charcoal samples that are unlikely to survive the ABOX process. The pretreated material was sealed in quartz combustion tubes and combusted at 900 °C, as described elsewhere [54]. Evolved carbon dioxide was cryogenically purified and collected, and its yield determined prior to conversion to graphite following standard graphitization procedures [55].

The ostrich shell samples, after visual inspection for the absence or for the presence of any powdery, potentially extraneous deposition, went through the rigorous cleaning [54]. Namely, shell surfaces were physically cleaned by abrasion of 10 to 25 percent of thickness with a Dremel^®^ tool followed by chemical etching of another 10% with 0.5M HCl for 1–5 minutes under sonication at room temperature. Following that the samples were rinsed in Milli-Q and oven dried. Hydrolysis was performed with concentrated H_3_PO_4_ acid in acid pre-cleaned glassware. First ~25% of evolving CO_2_ were also discarded to eliminate any possible surface diagenesis contamination. The remaining evolving CO_2_ was collected and converted to graphite [55]. Graphite targets were pressed into aluminum cathodes, and carbon isotopic ratios were measured on accelerator mass spectrometry installation STAR at ANSTO [56]. Measurement results were corrected for possible contamination in processing using the standard ANSTO laboratory blank correction procedure, which includes a number of test samples from radiocarbon free material (commercial and natural graphite, IAEA-C1 reference material [Marble]), processed following the same protocol as the studied samples, through to AMS targets, and measured together with unknown cathodes. Stable isotope measurements for δ^13^C isotopic fractionation correction were performed on a separate elemental analyser Elementar vario MICRO cube coupled to a Micromass Isoprime IRMS on residual graphite material after AMS analyses.

Results are plotted using the R statistical software [57] and packages of *ggplot2* [58], *knitr* [59], *rgl* [60], *RColorBrewer* [61], and *car* [62].

## Results

**Fig 5:**
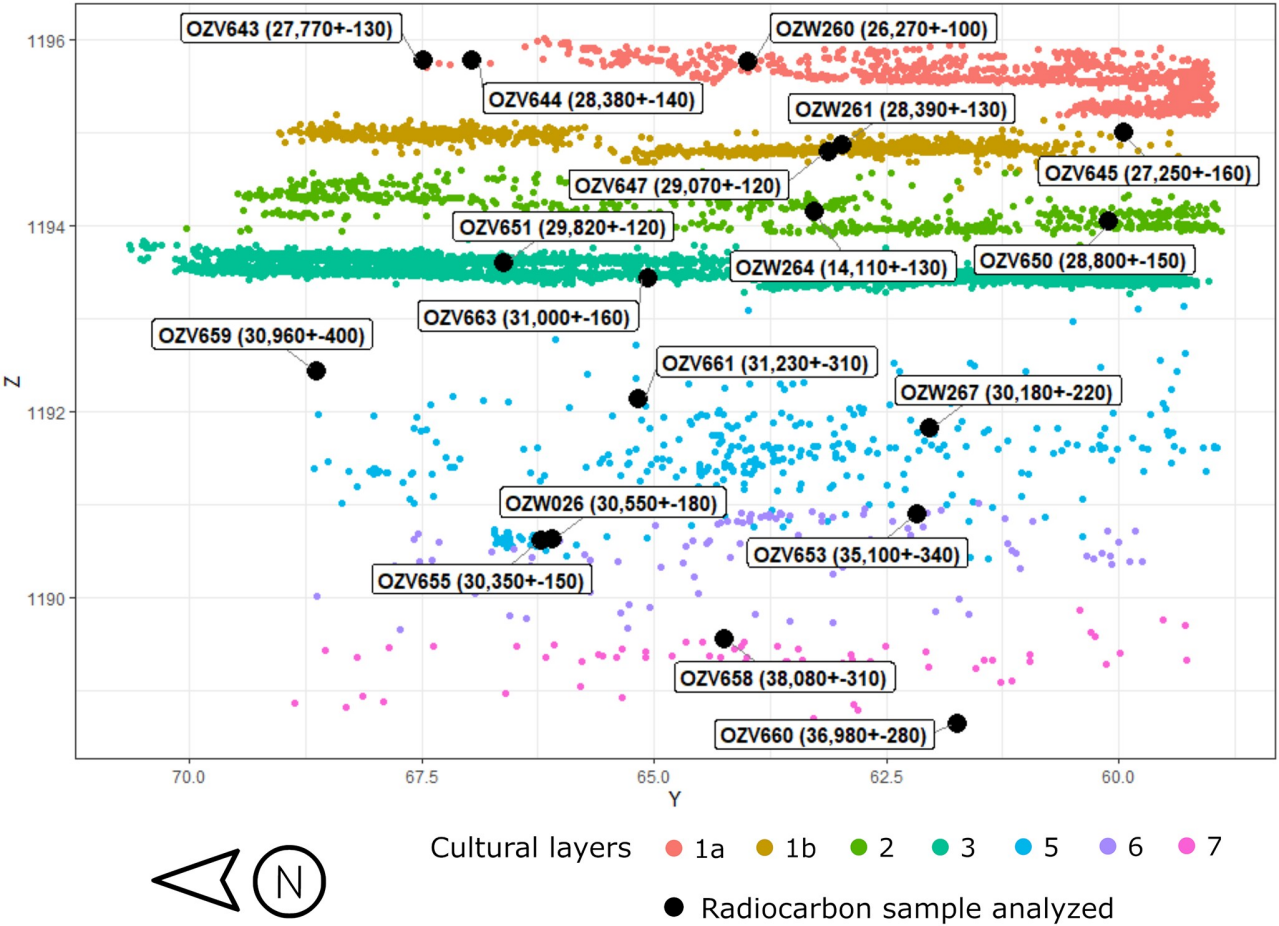
Spatial positions of the new radiocarbon dates from T3 of SDG2. Sample positions superimposed over the distribution of archaeological finds at T3 from an east-looking-west perspective. The Y coordinates (x axis) is based on the local site grid; the Z coordinates (y axis) reflects elevation in meters above mean modern sea level.

**Fig 6:**
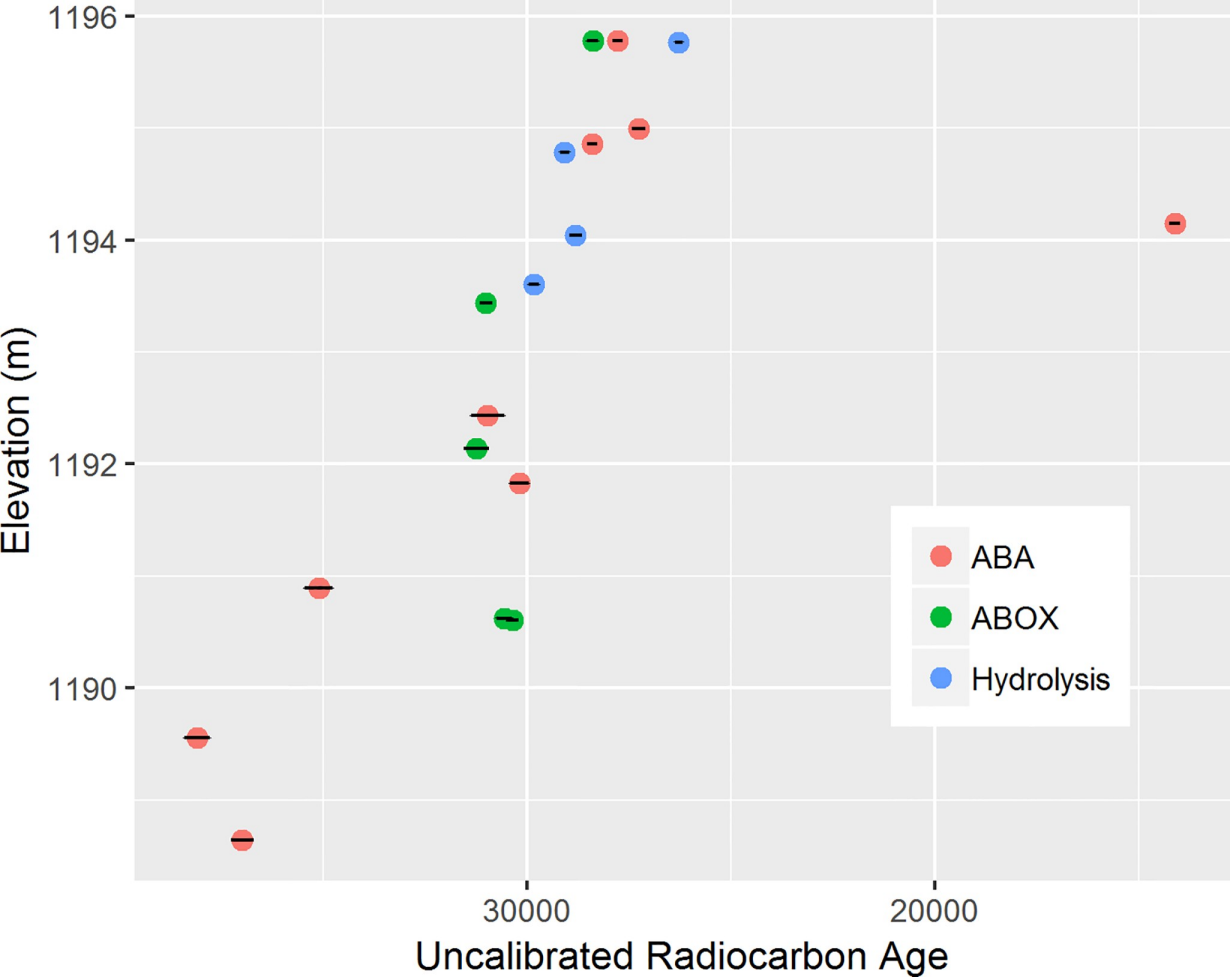
Age-depth relationship of the new radiocarbon dates from T3 of SDG2. The width of the black lines represents the error ranges at 1σ.

Out of the 32 samples investigated, 18 samples successfully produced age estimates (Table 1). The locations of these samples in relation to the CLs are shown in Fig 5 (the sample locations can be visualized three-dimensionally in S2 Appendix). The remaining 14 samples did not produce sufficient charcoal after pretreatment. Overall, the dates show a good age-depth relationship, except for one date associated CL2 that is significantly younger (Fig 6). Two of the charcoal samples corresponding with CL5, OZV655 and OZW026, came from a greater depth than that of OZV653, which was obtained from the underlying sediment associated with CL6. This is because, as mentioned earlier, the contact between the CL5 and CL6 sediment bodies is slopped (as can be seen in Fig 3), and the two CL5 samples came from a part of the site where the stratigraphic boundary is lower. In general, the dates from charcoal and ostrich eggshell are comparable. While the ABOX dates are generally slightly older than their ABA counterparts, the difference appears to be quite small.

**Table 1:** New radiocarbon dates from T3 of SDG2.

Unit	Material	Context Association	Lab ID	Pre-treatment	δ (_13_C) per mil	% Modern Carbon ± 1σ error	14C date (BP)	1σ Error
CL1a	OES	Scattered	OZW260	Hydrolysis	-11.3 ± 0.2	3.80 ± 0.04	26,270	100
CL1a	Charcoal	Scattered	OZV643	ABA	-25.0*	3.15 ± 0.05	27,770	130
CL1a	Charcoal	Scattered	OZV644	ABOX	-17.1	2.92 ± 0.05	28,380	140
CL1b	Charcoal	Scattered	OZV645	ABA	-24.6	3.36 ± 0.07	27,250	160
CL1b	Charcoal	Scattered	OZW261	ABA	-22.0 ± 0.1	2.92 ± 0.05	28,390	130
CL1b	OES	Scattered	OZV647	Hydrolysis	-12.7 ± 0.1	2.68 ± 0.04	29,070	120
CL2	OES	Scattered	OZV650	Hydrolysis	-12.7 ± 0.1	2.77 ± 0.05	28,800	150
CL2	Charcoal	Scattered but close to combustion feature	OZW264	ABA	-25.0*	17.27 ± 0.28	14,110	130
CL3	Charcoal	Scattered	OZV663	ABOX	-19.7 ± 0.1	2.11 ± 0.04	31,000	160
CL3	OES	Scattered but close to combustion feature	OZV651	Hydrolysis	-13.1 ± 0.1	2.44 ± 0.04	29,820	120
CL5	Charcoal	Scattered	OZW267	ABA	-23.5	2.33 ± 0.06	30,180	220
CL5	Charcoal	Scattered but close to combustion feature	OZV659	ABA	-25.0*	2.12 ± 0.10	30,960	400
CL5	Charcoal	Scattered	OZV661	ABOX	-25.0*	2.05 +- 0.08	31,230	310
CL5	Charcoal	From within combustion feature	OZV655	ABOX	-23.3 ± 0.1	2.29 +-0.04	30,350	150
CL5	Charcoal	From within combustion feature	OZW026	ABOX	-22.7	2.23 +- 0.05	30,550	180
CL6	Charcoal	Scattered	OZV653	ABA	-23.3 ± 0.1	1.27 +- 0.05	35,100	340
CL7	Charcoal	Scattered	OZV658	ABA	-22 ± 0.3	0.87 +- 0.03	38,080	310
Gravel	Charcoal	Scattered	OZV660	ABA	-25.0*	1.00 +- 0.03	36,980	280

δ (_13_C) determined by Elemental analysis isotope-ratio mass spectrometry (EA-IRMS).

* The value is assumed. A measured value is not available.

In order to interpret our dating results and to incorporate information from the stratigraphic contexts, we constructed a Bayesian chronological model by using the OxCal 4.3 software [35]. All ^14^C ages are calibrated against IntCal13 [36] with the *R_Date* command. We model the sequence in terms of the cultural layers as they are the units in which the dating samples were collected. Because the definition of the cultural layers does follow the site stratigraphic sequence, they provide a useful unit for constructing the site chronology when clearly observable geological units are lacking at the site. Each cultural layer was modelled as a *Phase*. Within each *Phase*, the age estimates are assumed to be unordered and evenly distributed. The *Phases* are then arranged into a *Sequence* based on their stratigraphic position. Note that when a cultural layer only has one age determination, which is the case for CL6, CL7 and the basal gravel layer, the date is inserted directed into the sequence without the need to specify the unit as a *Phase*. *Boundaries* are added in between *Phases* to estimate the age probability distribution between adjacent cultural layers. For the upper four cultural layers where the artifact concentrations are separated by sediment bodies with limited to no archaeology, the boundaries represent the ‘cultural gaps’ between the cultural layers. For the lower three cultural layers, the boundaries denote the transitions between the stratigraphic units associated with the cultural layers.

As mentioned earlier, the depositional environment of SDG2 shifted from a lake marsh to a lakeshore setting [31]. Based on sediment characteristics and the stratigraphic contact, this shift likely occurred at T3 between CL6 and CL5. The measured dates from these two layers suggest a gap of ~5,000 ^14^C years, indicating a possible pause in the sedimentation process or an erosional disconformity. To test for this possible phenomenon, we included two boundaries to demarcate the end of CL6 and the start of CL5 respectively with separate age probability distributions.

A *General t-type Outlier Model* is used to evaluate the presence of outliers among the measured dates [63]. Note that we did not use a *Charcoal Outlier Model* because it is designed specifically to capture the phenomenon of in-build age among charcoal samples. As such, the Charcoal Model follows a prior exponential distribution, meaning outliers can only be older than the modelled date [63]. Here, we argue that the effect of charcoal in-build age is minimal given the time range we are dealing with. Instead, by using a General Outlier Model that allows outliers to be either older or younger than the modelled date, we are better able to capture inconsistencies among the measured ages. Following Bronk Ramsey [63], each date is assigned a prior outlier probability of 5%, meaning we assume that every age estimate has a 1 in 20 chance of needing to be shifted in some way. During model construction, the posterior probability for the measurement being an outlier is determined, and the date is down-weighed accordingly in the modeling process. For example, if a date is found to have a posterior outlier probability of 30%, it means that the date is only included in 70% of the modeling iterations for generating the final chronological model. The OxCal code used here is provided in S3 Appendix.

**Fig 7:**
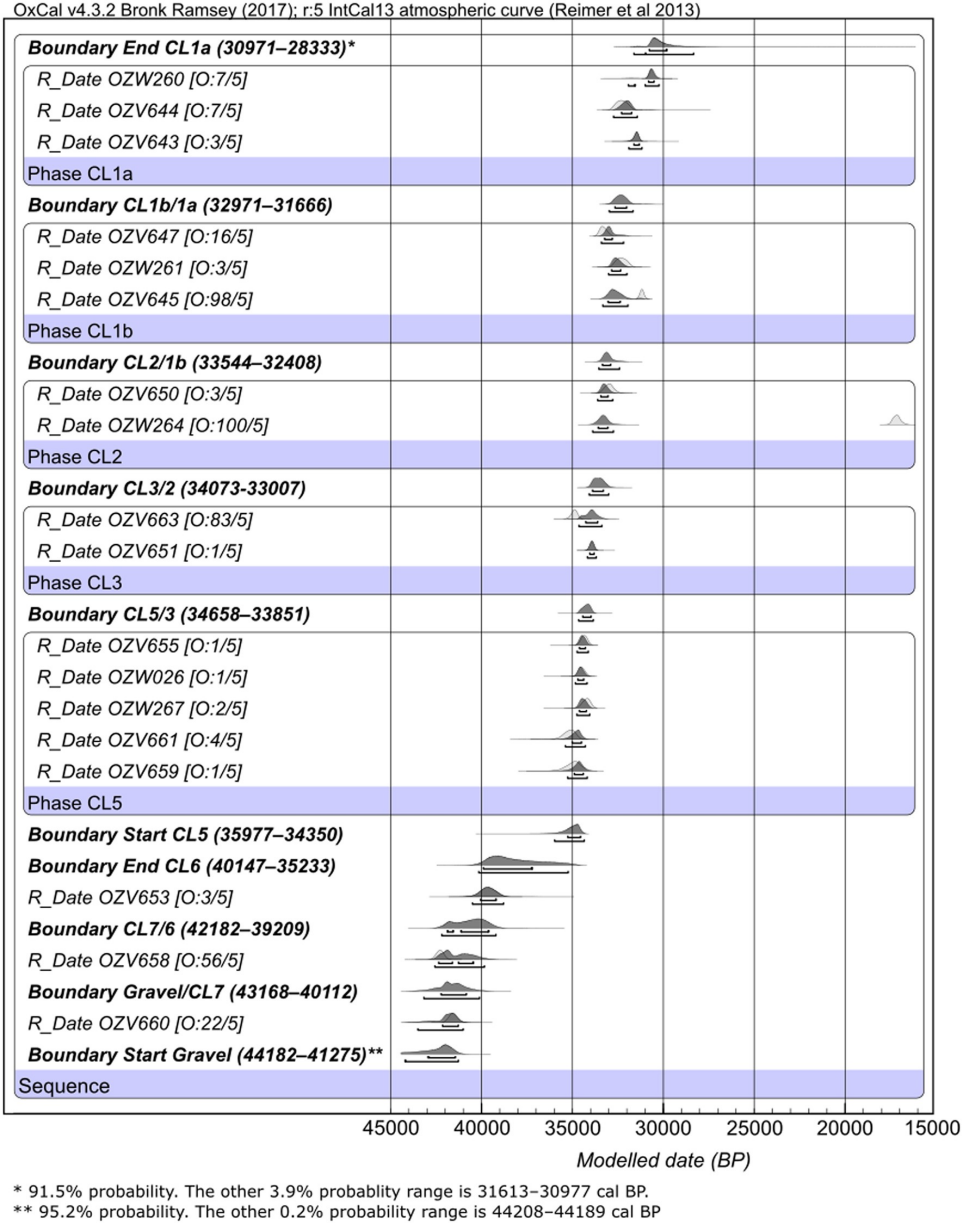
Bayesian model of the radiocarbon dates from T3 of SDG2. Light gray probability distributions depict the calibrated, unmodelled dates; dark gray distributions represent the modelled dates. The two brackets beneath each age distribution represent the 68.2% and 95.4% probability ranges. For each modelled date, the prior and posterior outlier probabilities are shown in brackets [O: posterior/prior] following the sample name on the left. For each modelled boundary, the modelled 95.4% probability age range in cal BP is listed following the boundary name. Model output table is provided in S2 Table.

Fig 7 shows the Bayesian chronological model. Three samples, OZV645, OZW264, and OZV663, have high posterior outlier probability (>80%) and are identified as outliers in our result. Interestingly, OZW264 was the only sample that produced an age that is magnitudes away from the rest of the measured dates. Yet, during sample collection, this charcoal was deemed to be of a more secure context as it was recovered close to a combustion feature. Because these samples are all scattered charcoal found outside of combustion features, it is plausible that their outlier ages reflect mixing events caused by localized bioturbation activities. However, it is difficult to identify the cause of these discrepancies at this point.

Our model suggests that human activities at SDG2 began ~43–40 ka cal BP (start of CL7) when the site was a lake marsh. At ~40–36 ka cal BP (end of CL6), the depositional environment of SDG2 started to change to a terrestrial setting, and yellow silt began to be lain under a low energy lake shore setting at ~36–34 ka cal BP (start of CL5). The two boundaries in between CL5 and CL6 produced overlapping probability distributions, which does not support our earlier suspicion that there was a temporal gap during the transition between CL6 and CL5. However, this negative result may reflect the disparity in the number of dated samples between the two layers, which could have caused the model to estimate a much wider age range for the end of CL6. From ~36 ka cal BP onwards, sediment accumulated relatively quickly until 28 ka cal BP, with over 5 m of loess-like silt deposited within 5,000–8,000 calendar years. Overall, the distribution of the archaeological materials deposited during this time is concentrated horizontal bands (CL1–3), suggesting that artifacts were discarded mainly during periods of stable land surfaces and subsequently buried via episodes of rapid sedimentation. Further sedimentary analysis and a new geoarchaeological study that include micromorphology are in the process of being carried out to clarify the nature of the depositional environments and site formation processes at SDG2.

## Discussion

This study is the first to conduct a systematic dating analysis of the entire archaeological sequence at SDG2. Our age determinations share a good age-depth relationship and are consistent with their respective stratigraphic positions. Previous dating studies of the site faced issues of uneven sample distribution among the Cultural Layers, age inversions, notable outliers, and discrepancies between dating techniques. The Bayesian chronological model reported here has limited outliers and thus represents a reliable estimate of the SDG2 archaeological sequence, providing an independent set of systematic age estimates for validating previous dating outcomes. We note that our ages for the upper layers of CL1a, CL2 and CL3 are older than previous estimates by ~2000 to 3000 calendar years. This difference may be due to the use of more stringent pre-treatment protocols in this study. If this is the case, it would imply that the previously published ages for these layers were underestimated due to contamination of modern carbon. In fact, according to Wood [64], samples of a similar age to these layers (~30 ka) would have an age underestimation in the observed range of 2000–3000 ^14^C years with just 1% of modern carbon contamination. Thus, future dating studies in this area would need to address the contamination issue by adopting appropriate sample selection strategies and stringent pretreatment protocols. The Bayesian model suggests that the SDG2 sedimentary sequence deposited in two major phases. The first phase occurred between CL7 and CL6 at 43–35 ka Cal BP above the basal gravel layer, followed by a second and more rapid phase from ~35–28 that span CL5 to CL1. This outcome supports previous interpretation of a shift in the depositional environment between CL6 and CL5 from a lacustrine to a terrestrial setting [31]. Based on stratigraphic comparison, Li et al. [33] placed this environmental shift between 33 and 34 ka cal BP. In contrast, our age model suggests that the lacustrine phase of the site history ended at 40–35 ka cal BP instead.

Comparing to the existing ages for SDG1, our results fall within the general timeframe that has been proposed for the Pleistocene context of the site, which ranges between 41–20 ka cal BP based on ^14^C and 16–46 ka based on OSL. However, it is important to note that the stratigraphic correlation between SDG1 and SDG2 remains unclear, and the two sites could very well have distinct site formation histories. In contrast to the over 12 m thick Pleistocene deposit at SDG2, the Pleistocene accumulation at SDG1 is limited to ~7 m in thickness and is intermitted with sediment layers comprised of fine sand that are absent at SDG2. In addition, the Pleistocene layer at SDG1 is capped by a 6.5 m thick Holocene deposit, whereas Holocene materials are largely absent at SDG2. This disparity in stratigraphic context means that it would be inappropriate to draw conclusions about the Pleistocene chronology at SDG1 using the dating results reported here.

However, our results here do have implication for the timing of the IUP at SDG. As mentioned earlier, two cores with IUP-like blade features were identified from the lithic assemblages of CL7 and CL5 at SDG2 [33]. The results of this study would place these cores at 43–39 ka cal BP and 36–34 ka cal BP respective. At face value, these ages support the interpretation that an IUP-like macroblade technology appeared in the SDG region by ~43–41 ka BP [8,33], placing it younger than the early IUP sites in the Altai (~47–45 ka) and northern Mongolia (~45 ka) [21,65]. Our results thus support the hypothesis that the IUP spread through to northern Mongolia and potentially the Lake Baikal region before arriving at north China. Yet, it is worth pointing out that, because the sedimentary layer associated with CL7 at SDG2 represents a lake marsh deposit, the CL7 lithic assemblage could be a mixture of reworked material rather than in situ discard. If it is the former, then the association between our age estimates, which is based on a floating charcoal sample, and the lithic artifacts in this context becomes questionable. This issue warrants future research to systematically date the basal lacustrine deposit at SDG2 by applying OSL and ^14^C analysis on sediment cores.

Additional studies should also consider more closely the stratigraphic transition between CL6 and CL5 at SDG2. While sedimentary and preliminary archaeobotanical analyses indicate the presence of water bodies nearby the site during MIS3 [31], the nature and extent of these water bodies and systems remain unknown. Quaternary environmental studies indicate that the climate of northwestern China was wetter ~41–30 ka, likely caused by a change in summer monsoon and the strong influence of north hemispheric westerly winds [66]. This wetter condition led to the development of extensive lakes in China’s deserts during the Late Pleistocene [67,68]. For instance, Zhang et al. [68] reported evidence for the proximity of paleolakes as well as other semi-connected and isolated water bodies in the Tengger Desert and the Badanjilin (Badain Jaran) Desert during MIS 3/2 that covered an area of more than 20,000 km^2^. The lacustrine deposit at the base of the SDG2 sequence, which is dated to 44–35 ka by our age estimates, could be related to a broader development of water bodies in the landscape during this period. These water systems would have had been an important factor in attracting early humans to the region. Moreover, the timing of the eastward spread of the IUP to China likely took place during mid-late MIS 3. The wetter condition of this period and the existence of lake and river systems across the historical ‘Hexi corridor’ could have provided an important ecological setting and pathways that facilitated the spread of human groups through an otherwise harsh arid environment along the modern Gobi Desert [21,22,25,69].

However, it is important to note that the timing for these Pleistocene water bodies in northwest China remains debated. In particular, there is a discrepancy between radiocarbon ages and OSL ages in the dating of the high lakestands of these paleolakes [70]. While ^14^C chronologies generally place the lake highstands at MIS 3, OSL analyses of shoreline deposits tend to suggest that the highstands took place earlier in MIS 5 [71–74]. While part of this disagreement may be related to the issue of ‘association’ (that is, what is the association between the dated event by the two methods and the phenomenon in question?), the causes for the inconsistency between the two dating methods in this setting remains unclear. More detailed study of the lacustrine deposits at SDG2 could help clarify this issue. Along with more refined paleoenvironmental data of the SDG2 region, these future research are essential to clarify the ecological setting of the IUP dispersal and the adaptations required for occupying these regions by early human species.

## Conclusion

The timing of the Late Paleolithic sequence at SDG has been contentious due in part to the identification of IUP-like stone artifacts. To date, the debate has largely focused on the chronology associated with artifacts identified with possible IUP features, including Levallois-like cores (or flat-faced cores), at sites such as SDG2. In this study, systematical sampling and analyses of charcoal and ostrich eggshell for radiocarbon dating have allowed the construction of a Bayesian chronological model for the SDG2 Late Paleolithic sequence. The model agrees with the general age range of SDG2 published previously and, more importantly, provides a higher-resolution chronological sequence that corroborates with existing stratigraphic and paleoenvironmental data of the site. Our findings are in line with existing chronological models for the IUP in north China at ~41 ka, and supports the spread of the IUP from Altai to northern Mongolia before moving south to China. The results here also demonstrate that, despite unfavorable preservation conditions for organic materials and small sample sizes, good age estimations can be produced by careful sample selection and thorough pretreatment. Importantly, the dates from the ostrich eggshell samples are in agreement with the charcoal ages. Ostrich eggshell preserves well in the SDG2 environment and thus could provide an important means for future age determinations of other Paleolithic sites in the region.

## Supporting information

S1 Table Published dates from SDG2. Radiocarbon ages listed in radiocarbon years before present (1950); OSL ages listed in calendar years.
(DOCX)

S2 Table Bayesian model table output from OxCal for the new radiocarbon dates from SDG2. Dates are listed in years before present (1950).
(DOCX)

S1 Appendix OxCal code for plotting the published dates from SDG2. (TXT)

S2 Appendix Three-dimensional locations of the radiocarbon samples anlaysed from SDG2 (T3). The plotted color points represent the locations of all of the point-provenienced stone artifacts, bones and ostrich eggshells. Colors denote the Cultural Layers that the finds are associated with.
(HTML)

S3 Appendix OxCal code for modelling the new radiocarbon dates from SDG2 (T3). (TXT)
