# Supplementary material for: A chronological model for the Late Paleolithic at Shuidonggou Locality 2, North China
Source: PLoS One. 2020 May 27;15(5):e0232682. doi: 10.1371/journal.pone.0232682 (PMC7252617; doi:10.1371/journal.pone.0232682)
Supplement: S1 Table — Radiocarbon ages listed in radiocarbon years before present (1950); OSL ages listed in calendar years. (DOCX) [file pone.0232682.s001.docx]

**S1. Published dates from SDG2.** Radiocarbon ages listed in radiocarbon years before present (1950); OSL ages listed in calendar years.

| **Cultural Layer** | **Original unit** | **Context** | **Material** | **Dating method** | **Lab** | **Sample number** | **^14^C BP for ^14^C / calendar years for OSL** | **error 1**σ | **Cal. BP (2**σ) | **References** |
| --- | --- | --- | --- | --- | --- | --- | --- | --- | --- | --- |
| 1a | Stratum 4 | Excavated sample | Bone | AMS ^14^C | Beta Analytic | BA140136 | 23450 | 80 |  | Li et al. 2019 |
| 1a | Stratum 4 | Excavated sample | Bone | AMS ^14^C | Beta Analytic | BA140137 | 23320 | 70 |  | Li et al. 2019 |
| 1a | Stratum 4 | Excavated sample | Bone | AMS ^14^C | Beta Analytic | BA140138 | 23270 | 70 |  | Li et al. 2019 |
| 1a | Stratum 4 | Excavated sample | Bone | AMS ^14^C | Beta Analytic | BA140139 | 23690 | 70 |  | Li et al. 2019 |
| 1a | Stratum 4 | Excavated profile | Sediment | OSL | Institute of Earth Environment, Chinese Academy of Sciences | IEE1880(S2-1) | 20300 | 1000 |  | Liu et al. 2009 |
| 2 | Hearth 1 | Naturally exposed profile | Charcoal | AMS ^14^C | Beta Analytic | Beta132982 | 26350 | 190 | 31012-30203 | Madsen et al. 2001; Gao et al. 2002 |
| 2 | Hearth 2 | Naturally exposed profile | Charcoal | AMS ^14^C | Beta Analytic | Beta132983 | 25670 | 140 | 30339-29414 | Madsen et al. 2001; Gao et al. 2002 |
| 2 | Hearth 2 | Naturally exposed profile | OES | AMS ^14^C | Beta Analytic | Beta132984 | 26930 | 120 | 31207-30818 | Madsen et al. 2001; Gao et al. 2002 |
| 2 | Hearth 3 | Naturally exposed profile | Charcoal | AMS ^14^C | Beta Analytic | Beta134824 | 26830 | 200 | 31220-30699 | Madsen et al. 2001; Gao et al. 2002 |
| 2 | Hearth 4 | Naturally exposed profile | Charcoal | AMS ^14^C | Beta Analytic | Beta134825 | 25650 | 160 | 30365-29365 | Madsen et al. 2001; Gao et al. 2002 |
| 2 | Hearth 5 | Naturally exposed profile | Charcoal | AMS ^14^C | Beta Analytic | Beta146355 | 26310 | 170 | 30975-30204 | Madsen et al. 2001; Gao et al. 2002 |
| 2 | Hearth 7 | Naturally exposed profile | Charcoal | AMS ^14^C | Beta Analytic | Beta146357 | 29520 | 230 | 34125-33230 | Madsen et al. 2001; Gao et al. 2002 |
| 2 | Hearth 10A | Naturally exposed profile | Charcoal | AMS ^14^C | Beta Analytic | Beta146358 | 23790 | 180 | 28283-27572 | Madsen et al. 2001; Gao et al. 2002 |
| 2 | Stratum 6 | Excavated sample | OES | AMS ^14^C | Beta Analytic | Beta207935 | 28420 | 160 | 32978-31719 | Gao et al. 2008 |
| 2 | Stratum 6 | Excavated sample | Charcoal | AMS ^14^C | Beta Analytic | Beta207936 | 28330 | 170 | 32822-31620 | Gao et al. 2008 |
| 2 | Stratum 6 | Excavated sample | Charcoal | AMS ^14^C | Peking University | BA110217 | 26450 | 120 | 30996-30492 | Chen et al. 2012; Li et al. 2013 |
| 2 | Stratum 6 | Excavated sample | Charcoal | AMS ^14^C | Peking University | BA110218 | 30360 | 120 | 34656-34056 | Chen et al. 2012; Li et al. 2013 |
| 2 | Stratum 6 | Excavated sample | Charcoal | AMS ^14^C | Peking University | BA110219 | 25090 | 90 | 29441-28844 | Chen et al. 2012; Li et al. 2013 |
| 2 | Stratum 6 | Excavated sample | Charcoal | AMS ^14^C | Peking University | BA110220 | 26040 | 90 | 30707-29911 | Chen et al. 2012; Li et al. 2013 |
| 2 | Stratum 6 | Excavated sample | Charcoal | AMS ^14^C | Peking University | BA110221 | 2520 | 30 |  | Chen et al. 2012; Li et al. 2013 |
| 2 | Stratum 6 | Excavated sample | Charcoal | AMS ^14^C | Peking University | BA110226 | 895 | 30 |  | Chen et al. 2012; Li et al. 2013 |
| 3 | Stratum 8 | Excavated sample | Bone | AMS ^14^C | Peking University | BA110223 | 28290 | 110 | 32665-31655 | Chen et al. 2012; Li et al. 2013 |
| 3 | Stratum 8 | Excavated sample | Bone | AMS ^14^C | Peking University | BA110222 | 27190 | 100 | 31324-30965 | Chen et al. 2012; Li et al. 2013 |
| 3 | Stratum 8 | Excavated profile | Sediment | OSL | Institute of Earth Environment, Chinese Academy of Sciences | IEE1881(S2-2) | 27800 | 1400 |  | Liu et al. 2009 |
| 4 | Stratum 10 | Excavated profile | Sediment | OSL | Institute of Earth Environment, Chinese Academy of Sciences | IEE1882(S2-3) | 20500 | 1100 |  | Liu et al. 2009 |
| 4 | Stratum 10 | Excavated sample | Charcoal | AMS ^14^C | Peking University | BA110224 | 985 | 30 |  | Chen et al. 2012; Li et al. 2013 |
| 5 | Stratum 13 | Excavated profile | Sediment | OSL | Institute of Earth Environment, Chinese Academy of Sciences | IEE1883(S2-4) | 29200 | 2100 |  | Liu et al. 2009 |
| 5 | Stratum 13 | Excavated sample | Bone | AMS ^14^C | Peking University | BA110227 | 20280 | 70 | 24569-24108 | Chen et al. 2012; Li et al. 2013 |
| 6 | Stratum 15-Upper | Excavated profile | Sediment | OSL | Institute of Earth Environment, Chinese Academy of Sciences | IEE1884(S2-5) | 23600 | 2400 |  | Liu et al. 2009 |
| 6 | Stratum 15-Lower | Excavated profile | Sediment | OSL | Institute of Earth Environment, Chinese Academy of Sciences | IEE1885(S2-6) | 38300 | 3500 |  | Liu et al. 2009 |
| 7 | Stratum 16-Upper | Excavated profile | Peat | AMS ^14^C | Peking University | BA07940 | 29759 | 245 | 34351-33490 | Liu et al. 2009 |
| 7 | Stratum 16-Lower | Excavated profile | Wood | AMS ^14^C | Peking University | BA07943 | 36329 | 215 | 41475-40441 | Liu et al. 2009 |
| 7 | Stratum 16-Lower | Excavated sample | Wood | AMS ^14^C | Peking University | BA110228 | 980 | 30 |  | Chen et al. 2012; Li et al. 2013 |
|  | Stratum 17 | Excavated profile | Sediment | OSL | Institute of Earth Environment, Chinese Academy of Sciences | S2-7 | 19600 | 2500 |  | Liu et al. 2009 |
|  | Stratum 17 | Excavated profile | Sediment | OSL | Institute of Earth Environment, Chinese Academy of Sciences | S2-8 | 64600 | 3600 |  | Liu et al. 2009 |
|  | Stratum 17 | Excavated profile | Sediment | OSL | Institute of Earth Environment, Chinese Academy of Sciences | S2-9 | 72000 | 4900 |  | Liu et al. 2009 |
