# Supplementary material for: A chronological model for the Late Paleolithic at Shuidonggou Locality 2, North China
Source: PLoS One. 2020 May 27;15(5):e0232682. doi: 10.1371/journal.pone.0232682 (PMC7252617; doi:10.1371/journal.pone.0232682)
Supplement: S2 Table — Dates are listed in years before present (1950). (DOCX) [file pone.0232682.s002.docx]

**S5 Table. Bayesian model table output from OxCal 4.3 for the new radiocarbon dates from SDG2.** Dates are listed in years before present (1950).

| Name | Unmodelled (BP) | | | | |  |  | Modelled (BP) | | | |  |  | C |
| --- | --- | --- | --- | --- | --- | --- | --- | --- | --- | --- | --- | --- | --- | --- |
|  | from | | to | % | from | to | % | from | to | % | from | to | % |  |
| Boundary End 1a |  |  | | |  |  |  | 30769 29813 | 29826 29809 | 68.1 0.1 | 31613 30971 | 30977 28333 | 3.9 91.5 | 96.1 |
| R_Date OZW260 | 30765 | | 30482 | 68.2 | 30880 | 30314 | 95.4 | 30810 | 30509 | 68.2 | 31919 31563 30993 | 31568 31540 30237 | 2.2 0.1 93.1 | 99.4 |
| R_Date OZV644 | 32605 | | 31975 | 68.2 | 32835 | 31715 | 95.4 | 32297 | 31743 | 68.2 | 32733 | 31432 | 95.4 | 98.9 |
| R_Date OZV643 | 31604 | | 31334 | 68.2 | 31809 | 31215 | 95.4 | 31614 | 31325 | 68.2 | 31891 | 31175 | 95.4 | 99.7 |
| Phase 1a |  | |  |  |  |  |  |  |  |  |  |  |  |  |
| Boundary 1b/1a |  |  | | |  |  |  | 32655 | 32018 | 68.2 | 32971 | 31666 | 95.4 | 98.2 |
| R_Date OZV647 | 33515 | | 33130 | 68.2 | 33648 | 32922 | 95.4 | 33226 | 32804 | 68.2 | 33405 | 32188 | 95.4 | 98.2 |
| R_Date OZW261 | 32615 | | 32004 | 68.2 | 32831 | 31745 | 95.4 | 32840 | 32344 | 68.2 | 33012 | 32002 | 95.4 | 98.6 |
| R_Date OZV645 | 31286 | | 31067 | 68.2 | 31405 | 30958 | 95.4 | 33036 | 32371 | 68.2 | 33325 | 31947 | 95.4 | 94.6 |
| Phase 1b |  | |  |  |  |  |  |  |  |  |  |  |  |  |
| Boundary 2/1b |  |  | | |  |  |  | 33346 | 32896 | 68.2 | 33544 | 32408 | 95.4 | 98.3 |
| R_Date OZV650 | 33250 | | 32745 | 68.2 | 33479 | 32465 | 95.4 | 33435 | 33043 | 68.2 | 33611 | 32781 | 95.4 | 99.1 |
| R_Date OZW264 | 17385 | | 16980 | 68.2 | 17546 | 16730 | 95.4 | 33574 | 33052 | 68.2 | 33883 | 32742 | 95.4 | 99.1 |
| Phase 2 |  | |  |  |  |  |  |  |  |  |  |  |  |  |
| Boundary 3/2 |  |  | | |  |  |  | 33890 | 33300 | 68.2 | 34073 | 33007 | 95.4 | 99.5 |
| R_Date OZV663 | 35050 | | 34712 | 68.2 | 35267 | 34564 | 95.4 | 34264 | 33610 | 68.2 | 34637 | 33379 | 95.4 | 96.1 |
| R_Date OZV651 | 34046 | | 33804 | 68.2 | 34176 | 33688 | 95.4 | 34044 | 33805 | 68.2 | 34173 | 33689 | 95.4 | 99.9 |
| Phase 3 |  | |  |  |  |  |  |  |  |  |  |  |  |  |
| Boundary 5/3 |  |  | | |  |  |  | 34426 | 33975 | 68.2 | 34658 | 33851 | 95.4 | 97.6 |
| R_Date OZV655 | 34522 | | 34176 | 68.2 | 34680 | 34022 | 95.4 | 34613 | 34286 | 68.2 | 34741 | 34128 | 95.4 | 98.9 |
| R_Date OZW026 | 34697 | | 34320 | 68.2 | 34858 | 34127 | 95.4 | 34698 | 34378 | 68.2 | 34836 | 34197 | 95.4 | 99.4 |
| R_Date OZW267 | 34426 | | 34000 | 68.2 | 34641 | 33839 | 95.4 | 34613 | 34232 | 68.2 | 34752 | 34046 | 95.4 | 98.5 |
| R_Date OZV661 | 35444 | | 34805 | 68.2 | 35834 | 34564 | 95.4 | 35010 | 34500 | 68.2 | 35385 | 34288 | 95.4 | 99.5 |
| R_Date OZV659 | 35300 | | 34506 | 68.2 | 35743 | 34146 | 95.4 | 34891 | 34401 | 68.2 | 35261 | 34187 | 95.4 | 99.5 |
| Phase 5 |  | |  |  |  |  |  |  |  |  |  |  |  |  |
| Boundary Start 5 |  |  | | |  |  |  | 35257 | 34553 | 68.2 | 35977 | 34350 | 95.4 | 98.5 |
| Boundary End 6 |  |  | | |  |  |  | 39895 37223 37209 | 37240 37219 37203 | 68 0.1 0.1 | 40147 | 35233 | 95.4 | 99.1 |
| R_Date OZV653 | 40063 | | 39230 | 68.2 | 40460 | 38834 | 95.4 | 40046 39207 | 39213 39201 | 67.8 0.4 | 40497 | 38788 | 95.4 | 99.5 |
| Boundary 7/6 |  |  | | |  |  |  | 41881 41858 41558 41124 41109 39626 | 41878 41576 41554 41116 39632 39608 | 0.1 8.6 0.1 0.3 58.7 0.4 | 42182 | 39209 | 95.4 | 96.3 |
| R_Date OZV658 | 42504 | | 42065 | 68.2 | 42734 | 41854 | 95.4 | 42355 41265 40453 | 41603 40464 40447 | 37.8 30.2 0.2 | 42564 | 39843 | 95.4 | 93.3 |
| Boundary 8/7 |  |  | | |  |  |  | 42220 | 40835 | 68.2 | 43168 | 40112 | 95.4 | 94.1 |
| R_Date OZV660 | 41806 | | 41328 | 68.2 | 42030 | 41051 | 95.4 | 42142 | 41279 | 68.2 | 43499 | 41009 | 95.4 | 96.5 |
| Boundary Start 8 |  |  | | |  |  |  | 42946 42904 | 42941 41441 | 0.1 68.1 | 44208 44182 | 44189 41275 | 0.2 95.2 | 98.2 |
| Sequence |  | |  |  |  |  |  |  |  |  |  |  |  |  |
| U(0,4) | 3.99E-17 | | 4 | 68.2 | 3.99E-17 | 4 | 95.4 | 3.084 3.756 | 3.608 3.812 | 63.2 5 | 2.924 | 3.872 | 95.4 | 1.6 |
| T(5) | -1.135 | | 1.135 | 68.2 | -2.65 | 2.65 | 95.4 |  |  |  |  |  |  | 22.9 |
| Outlier_Model General |  | | | |  |  |  | -1584 -1526 -1508 -583 995 1017 1033 1048 1076 1877 15810 15821 15850 16431 16454 16495 | -1577 -1514 -591 -567 1007 1024 1042 1064 1870 1901 15815 15843 16423 16447 16479 16503 | 0.1 0.2 28.5 0.3 0.2 0.1 0.2 0.3 21.5 0.5 0.1 0.4 14.8 0.3 0.4 0.2 | -2466 -2321 -2236 237 2151 2201 15481 16803 | -2438 -2302 -185 2145 2188 2226 16795 16830 | 0.1 0.1 39.7 32.1 0.2 0.1 23 0.1 | 80.9 |
