# Supplementary material for: A chronological model for the Late Paleolithic at Shuidonggou Locality 2, North China
Source: PLoS One. 2020 May 27;15(5):e0232682. doi: 10.1371/journal.pone.0232682 (PMC7252617; doi:10.1371/journal.pone.0232682)
Supplement: S2 Appendix — The plotted color points represent the locations of all of the point-provenienced stone artifacts, bones and ostrich eggshells. Colors denote the Cultural Layers that the finds are associated with. (HTML) [file pone.0232682.s004.html]

Locations of radiocarbon samples analysed from SDG2 (T3)


# Locations of radiocarbon samples analysed from SDG2 (T3)

#### Sam C. Lin

#### 15 July 2019

Packages required: “knitr”, “rgl”, “RColorBrewer”, “car”

```
## Loading required package: carData
```

```
## wgl 
##   2
```

You must enable Javascript to view this page properly.
